# Early Results of Sarcomeric Gene Screening from the Egyptian National BA-HCM Program

**DOI:** 10.1007/s12265-012-9425-0

**Published:** 2012-12-12

**Authors:** Heba Sh. Kassem, Remon S. Azer, Maha S. Ayad, Sarah Moharem-Elgamal, Gehan Magdy, Ahmed Elguindy, Franco Cecchi, Iacopo Olivotto, Magdi H. Yacoub

**Affiliations:** 1 Magdi Yacoub Heart Foundation, Cairo, Egypt; 2 Pathology Department and Clinical Genomics Centre, Alexandria Faculty of Medicine, Alexandria, Egypt; 3 Pharmacology Department, Cairo Faculty of Medicine and College of Pharmacy, University of Sharjah, Sharjah, United Arab Emirates; 4 National Heart Institute, Cairo, Egypt; 5 Cardiology Department, Alexandria Faculty of Medicine, Alexandria, Egypt; 6 Department of Cardiology, Azienda Ospedaliero Universitaria Careggi, Florence, Italy; 7 Imperial College, London, UK; 8 Bibliotheca Alexandrina-Yacoub Molecular Genetics Laboratory, 4th Floor: BA HCM Molecular Genetics Lab., 116 Horreya Avenue, Shallalat, Alexandria, Egypt

**Keywords:** Sarcomeric genotyping, HCM genetics, Egypt

## Abstract

The present study comprised sarcomeric genotyping of the three most commonly involved sarcomeric genes: *MYBPC3*, *MYH7*, and *TNNT2* in 192 unrelated Egyptian hypertrophic cardiomyopathy (HCM) index patients. Mutations were detected in 40 % of cases. Presence of positive family history was significantly (*p* = 0.002) associated with a higher genetic positive yield (49/78, 62.8 %). The majority of the detected mutations in the three sarcomeric genes were novel (40/62, 65 %) and mostly private (47/62, 77 %). Single nucleotide substitution was the most frequently detected mutation type (51/62, 82 %). Over three quarters of these substitutions (21/27, 78 %) involved CpG dinucleotide sites and resulted from C > T or G > A transition in the three analyzed genes, highlighting the significance of CpG high mutability within the sarcomeric genes examined. This study could aid in global comparative studies in different ethnic populations and constitutes an important step in the evolution of the integrated clinical, translational, and basic science HCM program.

## Background

Evaluation of genetic basis of inherited heart muscle disease is a prerequisite for further population, phenotypic, and mechanistic studies. Hypertrophic cardiomyopathy (HCM) is a model of common genetic heart disease attracting the interest of cardiologists, geneticists, scientists, and the public. Public interest is usually temporarily raised following the diagnosis of HCM as the cause of sudden death of a young competitive athlete [1]. Different population studies reported the prevalence of HCM, based on left ventricular (LV) wall thickness greater than 15 mm, as 1:500 (0.2 %) [2, 3]. However, this frequency represents the tip of the iceberg and is probably an underestimate since it represents only the clinically manifest HCM and does not include the prehypertrophic stage represented by the genotype-positive/phenotype-negative cases [4]. Based on the reported prevalence in different populations, an estimate of at least 160,000 individuals is expected to be affected with HCM within the over 80 million population of Egypt [5].

Although the incidence of this disease appears to be constant in different parts of the world, its phenotypic and genetic features seem to be quite heterogeneous. Laboratory genetic analysis undertaken over the past two decades have detected several hundred mutations in genes encoding proteins within and associated with the sarcomere [6–8].

Why various clinical manifestations are found among those who share the same sarcomeric gene mutation remains unknown [9]. Factors that account for this variability are only beginning to unravel. Diversity of the causal genes and mutations are partly responsible, but modifier genes and environmental factors also play a role in the phenotypic expression of HCM [10, 11]. Extensive and continuous research in different populations with different genetic backgrounds is imperative to understand the heterogeneity of this disease.

Mutation in any of the sarcomeric genes (*MYBPC3*, *MYH7*, *TNNT2*, *TPM1*, *TNNI3*, *MYL2*, *MYL3*, *ACTC*, and *TTN*) is usually identified in approximately half of the genetically screened HCM index cases [12, 13]. However, the proportion of detection of a pathogenic mutation is population-dependent and the reported frequency ranged from >50 % in France [12] and Italy [14] to <30 % in Sweden [15]. The three most commonly involved sarcomeric genes in HCM as reported in several populations in Europe and USA are myosin binding protein C (*MYBPC3*), β-myosin heavy chain (*MYH7*), and cardiac troponin T (*TNNT2*) [6, 16]. It is, therefore, reasonable to consider screening of those three genes as a first line strategy for molecular characterization of HCM patients in Egypt.

HCM diagnosis is usually delayed till complications develop and is, therefore, considered among the neglected diseases particularly in the developing world. Establishment of tertiary centers, which provide state-of-the-art management, can highly benefit these patients and allow establishment of clinical registries for systematized research and better understanding of the pathophysiology of this common genetic cardiovascular disorder [1]. Establishing national programs is invaluable for sharing disease knowledge and creating networks for optimizing HCM care and encouraging research, hence, the initiative of establishing the BA HCM national program in Egypt aiming for clinical and molecular characterization of HCM among Egyptian patients through nationwide collaboration between clinical satellites extending from Alexandria in the north to Aswan in the south and all being linked to molecular genetics laboratory hosted in the Bibliotheca Alexandrina premises.

## Subjects and Methods

### HCM Patient Population

**Fig. 1:**
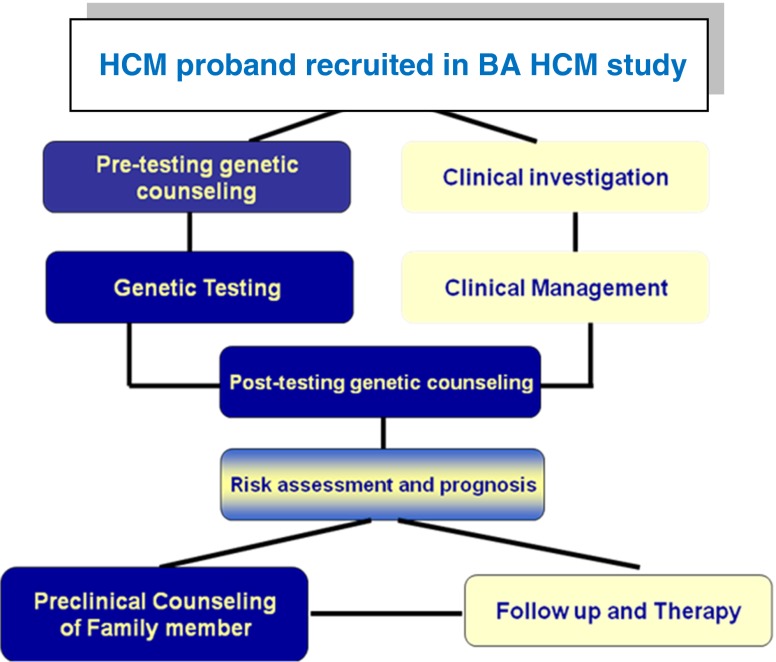
Multidisciplinary approach involving genetics and cardiology in management of HCM patients and their families in BA HCM program

The present study comprised sarcomeric (*MYBPC3*, *MYH7*, and *TNNT2*) genotyping of 199 consecutive unrelated HCM index patients recruited from the different geographic regions of Egypt from July 2007 to June 2010 following standard clinical evaluation by 2D echocardiography at the satellite HCM clinics in Alexandria Faculty of Medicine, Cairo Faculty of Medicine, National Heart Institute, and Aswan Heart Center. The study was approved by the Alexandria Faculty of Medicine and Aswan Heart Center Research ethics committees and informed written consent was obtained from all patients following provision of pretesting genetic counseling. HCM patients' clinical assessment and genetic testing in the BA HCM study is undertaken through a multidisciplinary approach involving genetics and cardiology specialties as illustrated in Fig. 1.

The diagnosis of HCM was based on the two-dimensional echocardiographic evidence of a hypertrophied, nondilated left ventricle (wall thickness ≥ 15 mm) in the absence of any other cardiac or systemic disease, including hypertension, capable of producing the magnitude of hypertrophy evident. The basic clinical and echocardiographic data was extracted from available patient clinical records and genotyping was undertaken blinded of patient clinical data. Detailed clinical characterization of the current patient cohort is beyond the scope of the current manuscript.

At least three generations of family pedigrees were constructed and cases were considered familial if there was a positive family history of clinically diagnosed HCM or considered suggestive to be familial through presence of family history of sudden cardiac death particularly if occurring at a young age <40 years. Also, history of consanguinity was always reported whenever present.

### Molecular Genetic Analysis

Genomic DNA was extracted from 4 to 6 ml of blood samples donated from index HCM patients following written informed consent and was amplified for the coding exons and flanking intronic sequences of the three candidate sarcomeric genes: *MYH7* (NM_000257.2, 38 coding exons were analyzed: third to 40th exons), *MYBPC3* (NM_000256.3, 34 exons, first to 34th), and *TNNT2* (NM_001001430.1, 16 exons, second to 17th). Primers sequences are available upon request.

Initial screening for mutations was undertaken using heteroduplex analysis by denaturing high-performance liquid chromatography (DHPLC) using WAVE™ DNA Fragment Analysis System. The conditions for DHPLC were developed on the basis of amplicon-specific melting profiles predicted by the Navigator software (Transgenomics, San Jose, CA, USA). Two melting temperatures were analyzed for most of the exons (54/88 = 61 %) and a single temperature for 24 (26 %) and three temperatures for 12 (13 %) amplicons. DHPLC sensitivity is reported as 95 % as compared to each amplicon-automatic sequencing [14].

**Fig. 2:**
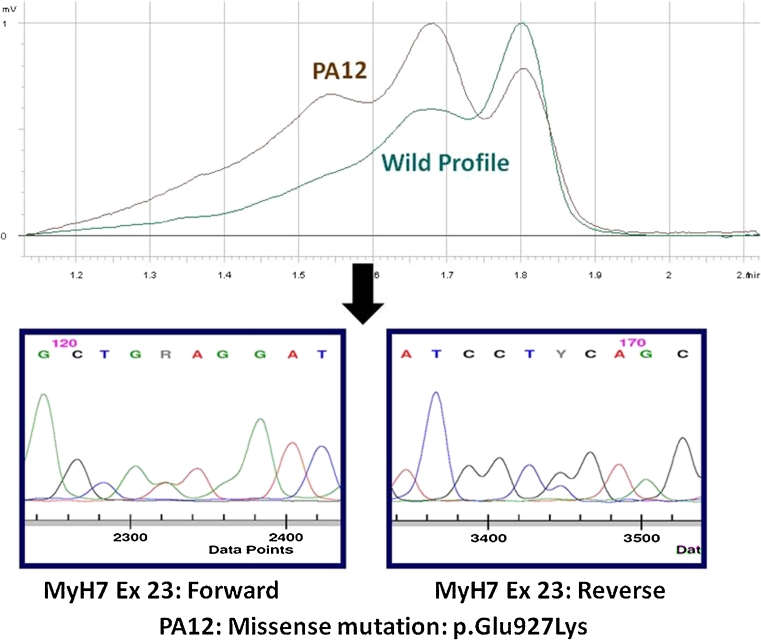
Strategy of genetic analysis in BA HCM study through initial screening with DHPLC (WAVE™ Transgenomics) followed by bidirectional automated sequencing of variant profile

Samples showing a variant profile (different from the wild pattern) for any of the amplicons were subjected to bidirectional sequencing using automated dye terminator cycle–capillary electrophoresis (ABI 3500 Applied Biosystems, Foster City, CA, USA) to determine the nature of the sequence change (Fig. 2).

Pathogenicity of variants detected in the present study was dependent on being previously reported to relate to HCM. For novel variants, pathogenicity was evaluated on several criteria including: absence in at least 200 unrelated chromosomes from age- and sex-matched healthy controls of the same ethnicity, cosegregation of the mutation with the disease phenotype in the proband's family, and the conservation of the mutated residue in different species in addition to pathogenicity prediction using in silico biosoftware analysis.

### In Silico Analysis of Variants Using Alamut-2.0.2 Biosoftware

#### Predicting Damaging Effect of Amino Acid Substitution

Prediction of novel missense mutation pathogenicity was undertaken through analyzing the physicochemical properties of the substituted amino acid relative to the wild one through licensed online predictive Alamut-2 Biosoftware (http://www.interactive-biosoftware.com).

#### Splice Site Error Predictions

Substitutions within introns flanking the coding exons of the three sarcomeric genes (*MYBPC3*, *MYH7*, and *TNNT2*) were firstly excluded as being reported as single nucleotide polymorphisms (SNPs) and were also tested in at least 200 alleles of healthy subjects (Appendix). The variants that were not detected in controls were subjected to in silico analysis for prediction of possible splice site alteration using online programs such as splicesitefinder (SSF) and Human Splicing Finder splice site analysis (HSF). The variants with a predicted possible splice site effect were considered as “variants of uncertain significance” and were checked whenever possible for cosegregation within the family with HCM phenotype to determine their potential for pathogenicity.

## Results

### Study Population

One hundred and ninety-nine patients were consecutively enrolled in the present study. The patient cohort comprised: 192 clinically diagnosed HCM patients, a single patient had LV noncompaction cardiomyopathy without hypertrophy (P76), and six patients were clinically suspected as HCM phenocopies: five patients were suspected to be infiltrative due to storage disease based on symmetrical hypertrophy and highly reflective myocardium (data not shown) and/or presence of history of parental consanguinity and presence of horizontal transmission pattern and one patient was suspected to have mitochondrial cardiomyopathy due to associated bilateral optic neuritis. Another patient was clinically suspected of LEOPARD syndrome due to associated pigmented cutaneous lesions. The fact that no further biochemical analysis was undertaken at the time of sarcomeric genetic testing, it was decided to include all enrolled patients to the three sarcomeric gene analyses. All suspected HCM phenocopies were negative for mutations in the three sarcomeric genes analyzed in the present study and were, therefore, excluded in further frequency estimation and statistical analysis.

Demographics of the 192 Egyptian HCM patients are shown in Table 1. Males were twice as common as females (68 % vs. 32 %), patients' age of presentation ranged from 2 to 70 years, and 53 % of the cases presented at or below 40 years. Cases were considered familial in the presence of positive family history of a diagnosis of HCM detected in 60 cases (60/192, 31 %) or presence of familial incidence of sudden cardiac death (18/192, 9 %). Sarcomeric mutations were detected in 62.8 % (49/78) of familial HCM cases. Summary of the basic parameters measured by 2D echocardiography and wave Doppler are shown in Table 1.

**Table 1:** Demographic data of the Egyptian hypertrophic cardiomyopathy patients

Feature	(%) N^c^ or mean ± SD
Gender	
Male	68 % (130/192)
Female	32 % (62/192)
Age at diagnosis (in years)	36.8 ± 16.80
Median (range)	38 (2–70)
≤40 years	53.1 % (102/192)
>40 years	45.8 % (88/192)
Family history	40.6 % (78/192)
Of diagnosed HCM	31.2 % (60/192)
Of sudden death	9.4 % (18/192)
+ve^a^ for sarcomeric mutation/+ve family history	62.8 % (49/78)
Clinical presentation	
Shortness of breath (NYHA)	
I	22.6 % (51/192)
II	44.3 % (85/192)
III	22.9 % (44/192)
IV	6.2 % (12/192)
Chest pain	65.6 % (126/192)
Palpitation	66.1 % (127/192)
Syncope/presyncope	52.6 % (101/192)
Baseline echocardiographic parameters	
Left atrium (mm)	44.7 ± 9.3
LV outflow gradient ≥ 30 mmHg	56.7 % (103/185)
Max. LV thickness (mm)	25.3 ± 19.7
<30 mm	80.9 % (149/184)
≥30 mm	19.0 % (35/184)
Symmetrical hypertrophy	1.1 % (2/185)
Asymmetrical hypertrophy	98.9 % (183/185)
Site of maximum thickness:	
Isolated septal hypertrophy	56.2 % (104/185)
Septal and posterior wall hypertrophy	28.7 % (53/185)
Apical hypertrophy	2.7 % (5/185)
LV end-diastolic dimension (mm)	44.2 ± 8.3
LV end-systolic dimension (mm)	26.6 ± 6.5
Mitral regurgitation (MR):	68 % (125/185)
Trivial/mild MR	47 % (87/185)
Moderate MR	14 % (26/185)
Severe MR	7 % (12/185)
SAM	62 % (114/185)
Major cardiac events^b^	
Sudden cardiac death	2 % (2/100)
Previous cardiac arrest	2 % (2/100)
Heart failure	3 % (3/100)
Stroke	1 % (2/100)
Interventions for obstruction/symptoms	
Alcohol septal ablation	1 % (2/192)
Surgical septal myectomy	27 % (52/192)
ICD	1 % (2/192)
Pacemaker	3 % (5/192)

*ICD* implantable cardioverter-defibrillator, *LV* left ventricular, *NYHA* New York Heart Association, *SAM* systolic anterior motion

^a^+ve: positive

^b^Total number includes cases with available follow-up data

^c^Total number varies according to available clinical data of parameters assessed

### Molecular Analysis

#### Prevalence of Mutations in *MYBPC3*, *MYH7*, and *TNNT2* Sarcomeric Genes

Seventy-seven index HCM patients harbored one or more mutation in any of the three analyzed sarcomeric genes (77/192, 40 %) (Tables 2 and 3). Among the genopositive patients, the highest frequency of mutations was detected in *MYBPC3* (48/77, ~60 %), followed by *MYH7* (24/77, ~30 %), and the least involved was *TNNT2* (8/77, ~10 %). Details of mutations detected are shown in Table 2 and potential splice site mutations predicted by in silico analysis are shown in (Table 3).

**Table 2:** Mutational spectrum in *MYBPC3*, *MYH7*, and *TNNT2* in Egyptian HCM cohort

Gene^a^	Ex	Mutation name In protein level	Mutation name in coding DNA level (cDNA)	Mutation type	Domain	Mode of mutation in CpG site	No. of patient (IDs)	Novelty N = novel, R = reported [Ref] (allele freq −EVS)^d^
*MYBPC3*	2	Val94Ala	c.281T > C	Missense	C0 (cardiac-specific region)	–	1 (P23)	N
*MYBPC3*	3	Pro102Leu	c.305C > T	Missense	Binding site to cardiac actin	–	2 (P19, PA69)	N
*MYBPC3*	4	Ser139X	c.416C > G	Nonsense	Binding site to cardiac actin	–	3 (P46, PA15, PU3)	N
***MYBPC3***	**5**	**Arg177His**	**c.530G > A**	**Missense**	**C1 (Ig-like C2-type 1)**	**CG > A**	**1 (P62)**	**(0.003)**
*MYBPC3*	5	Ala179GlnfsX59	c.534_541del	Frameshift	C1 (Ig-like C2-type 1)	NA	3 (PA25, PA52, PA46)	N
*MYBPC3*	5	Trp196X	c.587G > A	Nonsense	C1 (Ig-like C2-type 1)	–	1 (PA19)	N
*MYBPC3*	5	Trp196X	c.588G > A	Nonsense	C1 (Ig-like C2-type 1)	–	1 (PA18)	N
***MYBPC3***	**5**	**Ala216Thr**	**c.646G > A**	**Missense**	**C1 (Ig-like C2-type 1)**	**CG > A**	**1 (PA64)**	**R** [17]
*MYBPC3*	6	Glu258Lys	c.772G > A	Missense	MYBP-C motif (phosphorylation site)	CG > A	2 (PA59, PU10)	R [18]
*MYBPC3*	9	Ser296ThrfsX4	c.887del(G)	Frameshift	MYBP-C motif (phosphorylation site)	NA	1 (PA90)	N
*MYBPC3*	12	Glu319Ala	c.956A > C	Missense	MYBP-C motif (phosphorylation site)	–	1 (PA33)	N
*MYBPC3*	12	Tyr333X	c.999C > A	Nonsense	MYBP-C motif (phosphorylation site)	A < CG^c^	1 (P69)	N
*MYBPC3*	15	Cys436X	c.1308C > A	Nonsense	C2 (Ig-like C2-type 2)	A < CG^c^	1 (PA60)	N
***MYBPC3***	**15**	**Glu441Lys**	**c.1321G > A**	**Missense**	**C2 (Ig-like C2-type 2)**	**CG > A**	**4 (PA38, PA63, PU12, PA123)**	**R** [19]
*MYBPC3*	15	Thr445Met	c.1334C > T	Missense	C2 (Ig-like C2-type 2)	T < CG	1 (PA53)	N
*MYBPC3*	16	Arg470Pro	c.1409G > C	Missense	C3 (Ig-like C2-type 3)	CG > C^c^	1 (P55)	N
*MYBPC3*	16	Phe473_Glu474del	c.1418_1423del	In-frame	C3 (Ig-like C2-type 3)	NA	1 (PA80)	N
*MYBPC3*	17	Asp506ThrfsX7	c.1516del (G)	Frameshift	C3 (Ig-like C2-type 3)	NA	3 (P70, P62, P63)	N
***MYBPC3***	**17**	**Gly507Arg**	**c.1519G > A**	**Missense**	**C3 (Ig-like C2-type 3)**	**CG > A**	**1 (PA53)**	**R** [20]
*MYBPC3*	17	Asn515Asp	c.1543A > G	Missense	C3 (Ig-like C2-type 3)	–	1 (PA48)	N
*MYBPC3*	18	Ala558lysfsX9	c.1672_1673del	Frameshift	C4 (Ig-like C2-type 4)	NA	1 (P48)	N
***MYBPC3***	**19**	**Glu619Lys**	**c.1855G > A**	**Missense**	**C4 (Ig-like C2-type 4)**	**CG > A**	**2 (P48 ,P76)**	**R** [21]
*MYBPC3*	23	Ile769ThrfsX53	c.2306del(T)	Frameshift	C5 (Ig-like C2-type 5)	NA	1 (PA32)	N
***MYBPC3***	**24**	**Val771Met**	**c.2311G > A**	**Missense**	**C6 (fibronectin type-III 1)**	**CG > A**	**1 (PA26)**	**R** [22]
*MYBPC3*	25	Glu832Gly	c.2495A > G	Missense	C6 (fibronectin type-III 1)	–	2 (P4, P92)	N
*MYBPC3*	25	Arg845Pro	c.2534G > C	Missense	C6 (fibronectin type-III 1)	CG > C^c^	2 (P4, P92)	N
*MYBPC3*	27	Arg943X	c.2827C > T	Nonsense	C7 (fibronectin type-III 2)	T < CG	1 (PA87)	R [23]
*MYBPC3*	28	Leu993Phe	c.2977C > T	Missense	C8 (Ig-like C2-type 6)	–	1 (PA16)	N
*MYBPC3*	30	Trp1098X	c.3293G > A	Nonsense	*C9 (fibronectin type-III 3)*	–	4 (PA6, PA42, PA56,PA93)	R [24]
***MYBPC3***	**31**	**Arg1138Cys**	**c.3412C > T**	**Missense**	***C9 (fibronectin type-III 3)***	**T < CG**	**1 (PU11)**	**(0.0002)**
***MYBPC3***	**32**	**Glu1179Lys**	**c.3535G > A**	**Missense**	**Connection between C9 and C10**	**CG > A**	**1 (PA51)**	**R** [25]
*MYBPC3*	33	Glu1239del	c.3715_3717del	In-frame	C10 (Ig-like C2-type 7)	NA	1 (P39)	N
*MYBPC3*	33	Thr1256AsnfsX10	c.3766dup.(A)	Frameshift	C10 (Ig-like C2-type 7)	NA	1 (P36)	N
*MYH7*	6	Glu170Lys	c.508G > A	Missense	Myosin head	–	1 (P28)	N
*MYH7*	9	Arg249Gln	c.746G > A	Missense	Myosin head	CG > A	1 (PA55)	R [26]
*MYH7*	10	Leu267Val	c.799C > G	Missense	Myosin head	–	1 (PA26)	N
*MYH7*	11	Asp309Asn	c.925G > A	Missense	Myosin head	CG > A	1 (PA8)	N
*MYH7*	12	Glu379Lys	c.1135G > A	Missense	Myosin head	–	1 (P26)	N
*MYH7*	13	Asp394Glu	c.1182C > A	Missense	Myosin head	–	1 (PA54)	N
*MYH7*	14	Arg453Cys	c.1357C > T	Missense	Myosin head	T < CG	1 (P79)	R [27]
*MYH7*	15	Asn471Ser	c.1412A > G	Missense	Myosin head	–	1 (PA58)	N
*MYH7*	15	Gln498Arg	c.1493A > G	Missense	Myosin head	–	1 (PA13)	N
*MYH7*	18	Arg663Cys	c.1987C > T	Missense	Myosin head	T < CG	1 (P8)	R [28]
*MYH7*	19	Gly716Ala	c.2147G > C	Missense	Myosin head	–	1 (PA30)	N
*MYH7*	19	Arg719Gln	c.2156G > A	Missense	Myosin head	CG > A	2 (PA4, P67)	R [29]
*MYH7*	20	Pro731Leu	c.2192C > T	Missense	Myosin head	–	1 (P29)	R [30]
***MYH7***	**21**	**Ala797Thr**	**c.2389G > A**	**Missense**	**IQ domain**	**CG > A**	**1 (P16)**	**R** [31]
*MYH7*	22	Arg819Gln	c.2456G > A	Missense	Connection between head and coiled coil	CG > A	1 (P73)	N
*MYH7*	22	Lys847del	c.2539_2541del	In-frame	Myosin coiled coil	NA	1 (P53)	R [13]
*MYH7*	23	Glu927Lys	c.2779G > A	Missense	Myosin coiled coil	–	1 (PA12)	R [32]
*MYH7*	23	Met932Lys	c.2795T > A	Missense	Myosin coiled coil	–	1 (P56)	R [33]
*MYH7*	25	Glu1056Asp	c.3168G > C	Missense	Myosin coiled coil	–	1 (PA57)	N
***MYH7***	**35**	**Arg1662His**	**c.4985G > A**	**Missense**	**Myosin coiled coil**	**CG > A**	**1 (PU1)**	**(0.00008)**
*MYH7*	39	Ser1924AlafsX9	c.5769del (G)	Frameshift	Myosin coiled coil	NA	3 (P37, PA24, P85)	N
*TNNT2*	10	Arg92Trp	c.274C > T	Missense	Troponin T chain	T < CG	1 (P45)	R [34]
*TNNT2*	10	Arg92Gln	c.275G > A	Missense	Troponin T chain	CG > A	2 (P34, P98)	R [35]
*TNNT2*	11	Asn142Tyr	c.424A > T	Missense	Troponin T chain	–	1 (P22)	N
*TNNT2*	16	Asn262Asp	c.784A > G	Missense	Troponin T chain	–	1 (PA89)	N
*TNNT2*	16	Asn269Lys	c.807C > A	Missense	Troponin T chain	A < CG^c^	2 (P47, PA66)	N
*TNNT2*	17	Arg278Leu	c.833G > T	Missense	Troponin T chain	CG > T^c^	1 (PU7)	N

*Caps* and *italics* are names of genes according to HUGO nomenclature of genes

^a^GenBank reference: *MYBPC3*:NM_000256.3; *MYH7*: NM_000257.2; *TNNT2*: NM_001001430.1

^b^Uniprot reference: cardiac myosin-binding protein C: Q14896; myosin heavy chain 7: P12883; cardiac troponin T: P45379; IQ domain: calmodulin binding domain: http://www.uniprot.org/uniprot/Q14896,P12883,P45379

^c^CpG substitution by a mutagenic mutation other than deamination of methylated cytosine to thymine

^d^Allele frequency obtained from Exome Variant Server (EVS) of Exome Sequencing Project: http://evs.gs.washington.edu/EVS/) [36]. Mutations in bold were reported in EVS

**Table 3:** Possible splice site error associated with novel intronic substitution

Gene	Intron	Mutation name according to coding DNA level (cDNA)	No. of patients (IDs)	Cosegregation	Detection in Egyptian controls (200 alleles) or reported in EVS^a^ (allele freq) 1 = present 0 = Absent
*MYBPC3*	3	c.407 − 7C > A	2 (P14, P16)	Absent (P14′a)	0
*MYBPC3*	7	c.821 + 3G > T	1 (PU1)	Yes (PU1′A)	0
*MYBPC3*	16	c.1458 − 7C > A	1 (P89)	TBP*	0
*MYBPC3*	16	c.1458 − 17C > G	1 (P17)	TBP*	0
*MYBPC3*	22	c.2149 − 8C > T	1 (P100)	TBP*	0
*MYBPC3*	32	c.3627 + 2del	1 (PU2)	NT	0
***TNNT2***	**14**	**c.690 − 4G > T**	**1 (P32)**	TBP*	**1 (0.0002)** ^a^
*TNNT2*	15	c.781 − 48_64del	1 (PA78)	TBP*	0

*MYBPC3* NM_000256.3, *MYH7* NM_000257.2, *TNNT2* NM_001001430.1, *NT* not tested, TBP* To *B*e *P*ursued by family clinical screening to test cosegregation in affected family members

^a^Allele frequency obtained from Exome Variant Server (EVS) of Exome Sequencing Project (ESP): http://evs.gs.washington.edu/EVS/) [36]. Mutations in bold were reported in EVS

Missense mutation was the most commonly encountered mutation type in the three sarcomeric genes. There were 62 individual mutations detected in any of the three sarcomeric genes in the present cohort, the majority of which (47/62, 77 %) were detected in single index patient (i.e., private) (Tables 2 and 3). Most of the mutations detected in the studied cohort were novel (40/62, 65 %) and were not detected in 200 alleles of age-, sex-, and ethnically matched control series nor in the 1000 Exome Variant Server (EVS) [36]. Three missense mutations detected in our HCM patients (Ala177His, Arg1138 in MYBPC3, and Arg1662His in MYH7) were found in the EVS; however, they were reported at low allele frequencies (0.003, 0.0002, and 0.00008, respectively). Additionally, seven missense mutations previously reported as pathogenic in relevance to HCM and also found in the present cohort (Ala216Thr, Clu441Lys, Gly507Arg, Glu619Lys, Val771Met, Glu1179Lys in MYBPC3, and Ala797Thr in MYH7) were also reported in the EVS. This finding should be interpreted with caution. While on the one hand, these variants might be regarded as rare polymorphic nonpathogenic variants, on the other hand, it could still have a pathogenic and/or modifier effect in a disease with the heterogeneous nature of HCM having later age of onset and reduced penetrance especially when considering controls not thoroughly evaluated by electrocardiogram (ECG) and echo. Further screening in a larger number of ethnic-matched controls with thorough cardiologic evaluation is warranted.

##### *MYBPC3* Mutation Spectrum

*MYBPC3* was the most commonly involved in the present cohort (47 patients were *MYBPC3*-genopositive, 47/192, 24 %). Thirty-five individual mutations were detected in *MYBPC3*, 18 mutations were missense, seven were nonsense, eight were deletion mutation of which six caused frameshift effect resulting in premature stop codon and two mutations caused in-frame deletion of a single or more amino acids (Table 2).

**Fig. 3:**
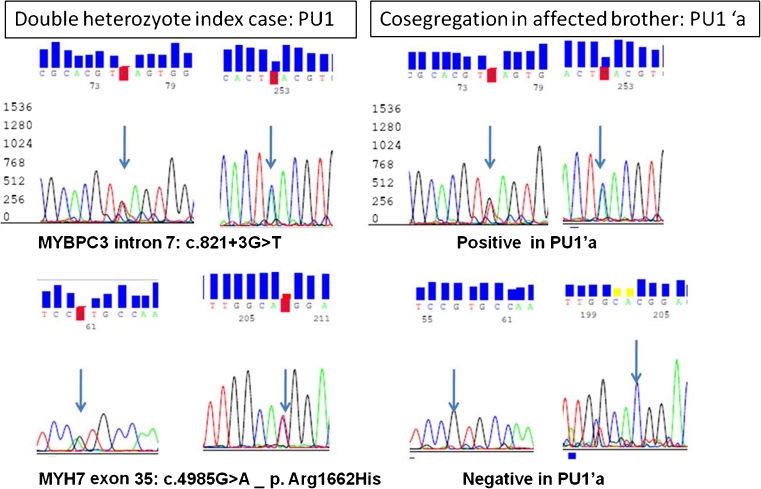
Bidirectional automated sequencing of *MYBPC3* exon7 and *MYH7* exon35 and flanking intronic sequences in index patient PU1 and screening of an affected brother (PU1′a) showing cosegregation of *MYBPC3* intron 7 substitution mutation: c.821 + 3G > T with potential splice effect as predicted by in silico tools and not *MYH7* missense variant (Arg1662His) indicating likely pathogenicity of the *MYBPC3* intronic variant

In addition, two mutations were detected in introns 32 and 7, namely, c.3627 + 2delT (PU2) and c.821 + 3G > T (PU1), respectively (Table 3). These variants are likely to result in splice site error since both were absent in 200 chromosomes of unrelated healthy subjects and the former is affecting the conserved 5′ donor splice site dinucleotide GT of intron 32 and the latter (c.821 + 3G > T) was predicted to result in splice error by in silico modeling and was found to cosegregate with the HCM phenotype in a HCM family member (PU1′a), thus, in favor of its potential pathogenicity. In further support of this speculation is the fact that the index patient PU1 was double heterozygote and also carried missense mutation in *MYH7* exon39 (p.Arg1662His) that did not cosegregate in the affected family member (PU1′a) (Fig. 3).

Twenty-five mutations (25/35, 71 %) of *MYBPC3* were private (i.e., reported in a single index patient) and ten mutations were found in more than one patient: five mutations were detected in two unrelated index patients, three mutations were individually found in three unrelated patients, and two mutations were detected in four unrelated index patients suggesting a possible founder effect (Table 2).

A single patient had noncompaction LV cardiomyopathy without hypertrophy (P76) and carried missense mutation in *MYBPC3* (p.Glu619Lys) that was also detected in another HCM patient in the present cohort and was previously reported in association with HCM [21].

##### *MYH7* Mutation Spectrum

Twenty-one individual mutations of *MYH7* were detected in 24 unrelated index patients (24/192, 12.5 %). Of these, 19 were missense, two were deletion mutations, one of which was in-frame deletion resulting in deletion of a single amino acid (Lys847del) and one resulted in frameshift mutation (p.Ser1924AlafsX9). All mutations were private except two that were detected in more than one index patient, Arg719Gln was detected in two and Ser1924AlafsX9 was detected in three unrelated index patients (Table 2).

##### *TNNT2* Mutation Spectrum

*TNNT2* was the least commonly involved gene in our cohort (8/192, 4 %). Six missense mutations were detected in *TNNT2*, most of which were novel (4/6, 67 %) (Table 2). In addition, two missense mutations involving Arg92 codon (Arg92Trp and Arg92Gln) [34, 35] were detected in one and two patients, respectively.

##### Occurrence of Double Hits

Seven patients in the present cohort (7/192, 4 %) carried double hit mutations in one or more of the analyzed sarcomeric genes. Five were compound heterozygotes within *MYBPC3* (P62, PA53, P48, P4, and P92). Two patients were double heterozygotes (PA26 and PU1): PA26 carried missense mutation in *MYBPC3* (p.Val771Met) and another in *MYH7* (Leu267Val) and PU1 had substitution mutation within intron 7 of *MYBPC3* (c.821 + 3G > T) with a predictive splice error effect and a missense mutation in *MYH7* (Arg1662His), and only the intronic variant was cosegregating in an affected family member (PU1′).

##### Possible Founder Effect

Two novel *MYBPC3* mutations showed a particular geographic distribution in Egypt indicating a possibility for a founder effect. The frameshift mutation p.Trp1098X in exon30 was detected in three unrelated patients from the northwest Mediterranean coast in Alexandria governorate inhabited by Bedouin tribes. Another frameshift mutation, p.Asp506ThrfsX7 in exon15, was detected in three unrelated index patients from the Nile River Delta region in Damietta governorate.

Interestingly, two unrelated index patients (P4 and P92) were compound heterozygotes for two mutations (p.Glu832Gly and p.Arg845Pro) within exons 25 and 26 of *MYBPC3* gene. These two mutations are likely to be in cis as they were cosegregating in an affected family member of P4. This may also suggest a founder effect due to the fact that both mutations were cosegregating in unrelated index patients.

##### Bioinformatic Analysis of Substitution Mutations for Possible Splice Error Effect

All novel variants detected in intronic sequences flanking the coding exons of the three sarcomeric genes were initially checked through online genomic SNP database (http://snpper.chip.org/). Those which were not determined as a SNP were subjected to in silico analysis for prediction of splice error effect (Table 3).

Apart from the single variant that involved the conserved donor splice site of intron 32 of *MYBPC3* (c.3627 + 2delT) in PU2 and was considered as pathogenic, nine intronic novel variants were predicted by in silico analysis to cause a splice site error. Three were determined as not likely to be pathogenic since two of which were detected in the Egyptian control series [c.2149-5C > T(P87) in intron 22 of *MYBPC3* (detected in 1 % of the controls) and c.3337-3dup (PA2) in intron 27 of *MYH7* (was found in 16 % of the controls series)], and one variant detected in *MYBPC3* intron 3 (c.407-7C>A) was not cosegregating with HCM phenotype in an affected relative of index P14.

Cosegregation provides a direct method for excluding that a variant is the sole cause of disease within a family. It was, therefore, always sought whenever possible. In the present study, c.821 + 3G > T in intron 7 of *MYBPC3* was cosegregating with HCM phenotype in a family member of index PU1, thus, providing evidence for potential pathogenicity, especially that the other mutation found in PU1 in *MYH7* (p.Arg1662His) was not cosegregating in the affected relative (Fig. 3). The missense variant (p.Arg1662His in MYH7) was reported in the EVS with a very low allele frequency: 0.00008 (Table 2).

Three intronic substitutions in *MYBPC3* (c.1458-7C > A, c.1458-17C > G, and c.2149-8C > T) and two in *TNNT2* (c.690-4G > T and c.781-48_64del) were classified as variants of undetermined clinical significance since they were predicted by in silico analysis to result in splice error (Table 3) and were absent in 200 chromosomes of healthy subjects. It is worth mentioning that variant c. 690-4G > T was reported in EVS at an allele frequency of 0.0002 and that cosegregation was not tested till writing of the current manuscript and should be pursued prior to considering the potential pathogenicity of this variant. It is worth mentioning that these in silico predictions of splice errors should be validated by ribonucleic acid (RNA) analysis.

##### In Silico Prediction of Novel Missense Mutation Pathogenicity

The novel missense substitutions are initially excluded in at least 200 chromosomes of matched healthy volunteers and are also subjected to in silico bioinformatic analysis for prediction of pathogenicity, which included several scoring systems (Table 4). The Grantham Difference, which depends on the composition, polarity, and molecular volume of the wild and substituted amino acid, shows that the higher the score, the less tolerated the amino acid substitution. Align GVGD combines Grantham difference of amino acids and protein multiple sequence alignments to predict whether the missense substitution falls in a spectrum from enriched deleterious to enriched neutral (http://agvgd.iarc.fr/). Sorting Intolerant from Tolerant (SIFT) depends on the degree of conservation of amino acid residues in sequence alignments derived from closely related sequences in different species (http://sift.jcvi.org). Polymorphism Phenotyping (Polyphen-2) predicts possible impact of an amino acid substitution on the structure and function of a human protein using physical and comparative considerations (http://genetics.bwh.harvarad.edu/pph2/).

**Table 4:** Bioinformatic analysis of novel missense mutations associated with hypertrophic cardiomyopathy among Egyptian patients

Gene/mutation in protein level	Grantham difference (0–225)	SIFT^b^ D = deleterious (score < 0.05) T = tolerated (score > 0.05)	Align GVGD class^c^	Polyphen-2 (score)^d^	Likelihood of pathogenicity^e^
*MYBPC3*/Val94Ala	64	D (0.01)	C25	Probably damaging (0.999)	Likely
*MYBPC3*/Pro102Leu^a^	98	D (0.04)	C0	Benign (0.008)	Pathogenic
*MYBPC3*/Arg177His	29	D (0.01)	C0	Possibly damaging (0.889)	Likely
*MYBPC3*/Glu319Ala	107	T (0.53)	C0	Benign (0.014)	Uncertain
*MYBPC3*/Thr445Met	81	D (0.00)	C65	Probably damaging (1.000)	Likely
*MYBPC3*/Arg470Pro	103	D (0.00)	C65	Probably damaging (0.999)	Likely
*MYBPC3*/Asn515Asp	23	D (0.00)	C15	Benign (0.040)	Uncertain
*MYBPC3*/Glu832Gly^a^	98	D (0.00)	C65	Probably damaging (1.000)	Pathogenic
*MYBPC3*/Arg845Pro^a^	103	D (0.00)	C65	Probably damaging (1.000)	Pathogenic
*MYBPC3*/Leu993Phe	22	D (0.03)	C0	Probably damaging (0.983)	Likely
*MYBPC3*/Arg1138Cys	180	D (0.00)	C65	Probably damaging (1.000)	Likely
*MYH7*/Glu170Lys	56	D (0.00)	C0	Probably damaging (0.996)	Likely
*MYH7*/Leu267Val	32	D (0.00)	C0	Benign (0.011)	Uncertain
*MYH7*/Asp309Asn	23	D (0.00)	C0	Probably damaging (0.963)	Uncertain
*MYH7*/Glu379Lys	56	D (0.00)	C0	Benign (0.315)	Uncertain
*MYH7*/Asp394Glu	45	D (0.00)	C0	Probably damaging (1.000)	Likely
*MYH7*/Asn471Ser	46	D (0.00)	C45	Benign (0.019)	Likely
*MYH7*/Gln498Arg	43	D (0.00)	C35	Benign (0.370)	Likely
*MYH7*/Gly716Ala	60	D (0.00)	C55	Possibly damaging (0.948)	Likely
*MYH7*/Arg819Gln	43	D (0.00)	C35	Probably damaging (1.000)	Likely
*MYH7*/Glu1056Asp	45	D (0.00)	C0	Probably damaging (0.997)	Likely
*MYH7*/Arg1662His	29	D (0.00)	C0	Benign (0.001)	Uncertain
*TNNT2*/Asn152Tyr	143	T (0.06)	C0	Possibly damaging (0.951)	Likely
*TNNT2*/Asn272Asp	23	T (0.32)	C0	Possibly damaging (0.820)	Uncertain
*TNNT2*/Asn269Lys^a^	94	T (1.00)	C0	Benign (0.028)	Pathogenic
*TNNT2*/Arg288Leu	102	T (1.00)	C0	Probably damaging (0.972)	Uncertain

^a^De novo concurrent with HCM in more than a single family

^b^Sorting intolerant from tolerant prediction

^c^Scores include GV = 0 (invariable), 0 < GV < 62 = variable conservative, GV > 62 = variable nonconservative

^d^Polymorphism phenotyping

^e^Likelihood of pathogenicity based on in silico analysis and de novo concurrence of variant in unrelated HCM cases

The likelihood of pathogenicity of a novel missense variant is analyzed according to output of the available bioinformatic tools. At least two programs agreeing in predictive output is used to determine pathogenicity. The concurrent occurrence of novel variants of uncertain clinical significance in several unrelated families such as *MYBPC3*/Pro102Leu (in P19 and PA69), *MYBPC3*/Glu832Gly and *MYBPC3*/Arg845Pro (in P4 and P92), and *TNNT2*/Asn269Lys (in P47 and PA66) in addition to their absence in our control series and in the 1000 exome project (EVS) [36] favored their pathogenic potential.

Uncertain and likely pathogenic variants were always checked for cosegregation whenever possible. Interestingly, the *MYH7*/Arg1662His detected in a single index patient (PU1) detected as a second hit was determined by in silico analysis as a benign/tolerated variant and was not cosegregating with HCM phenotype in an affected family member, and its being reported in EVS (with an allele frequency of 0.00008) are in favor of its less likelihood of a direct role in pathogenicity of HCM and may have a modifier effect. The de novo concurrence of the double in cis variants of *MYBPC3* exon25 (Glu832Gly) and exon26 (Arg845Pro) in two families (P4&P92) were found to cosegregate in the affected family member of P4 favoring the pathogenic effect of one or either of them.

##### Mutations Involving CpG Dincleotides

All substitution mutations were analyzed in context of the reference GenBank sequence of the three sarcomeric genes. It was observed that over half (27/51, 53 %) of all substitution mutations resulting in missense and nonsense effect occurred in CpG dinucleotides [60 % in *MYBPC3* (15/25), 40 % in *MYH7* (8/20), and 66.6 % in *TNNT2* (4/6)] (Table 2). In relation to mode of nucleotide substitution in the CpG site, it was observed that the most commonly encountered types were the G > A and C > T transitions, 55.6 % (15/27) and 22.2 % (6/27), respectively. On the other hand, the C > A, G > C, and G > T transversion types were detected less frequently, [11.1 % (3/27), 7.4 % (2/27), and 3.7 % (1/27), respectively] (Table 5). The fact that over three quarters of these single nucleotide substitutions involving the CpG sites (21/27, 78 %) resulted from C > T or G > A transition in the three genes [73.3 %(11/15) in *MYBPC3*, 100 % (8/8) in *MYH7*, and 50 % (2/4) in *TNNT2*] highlights the significance of CpG high mutability resulting from deamination of 5-methylcytosine to thymine within the CpG sites in sarcomeric genes causing HCM among Egyptian patients.

**Table 5:** Frequency of different substitution modes within CpG in the present HCM cohort

Mode of substitution in CpG	Frequency in present cohort
G > A	55.6 % (15/27)
C > T	22.2 % (6/27)
C > A	11.1 % (3/27)
C > G	0 % (0/27)
G > C	7.4 % (2/27)
G > T	3.7 % (1/27 )

#### Influence of Genetic Mutation on Clinical Phenotype

**Fig. 4:**
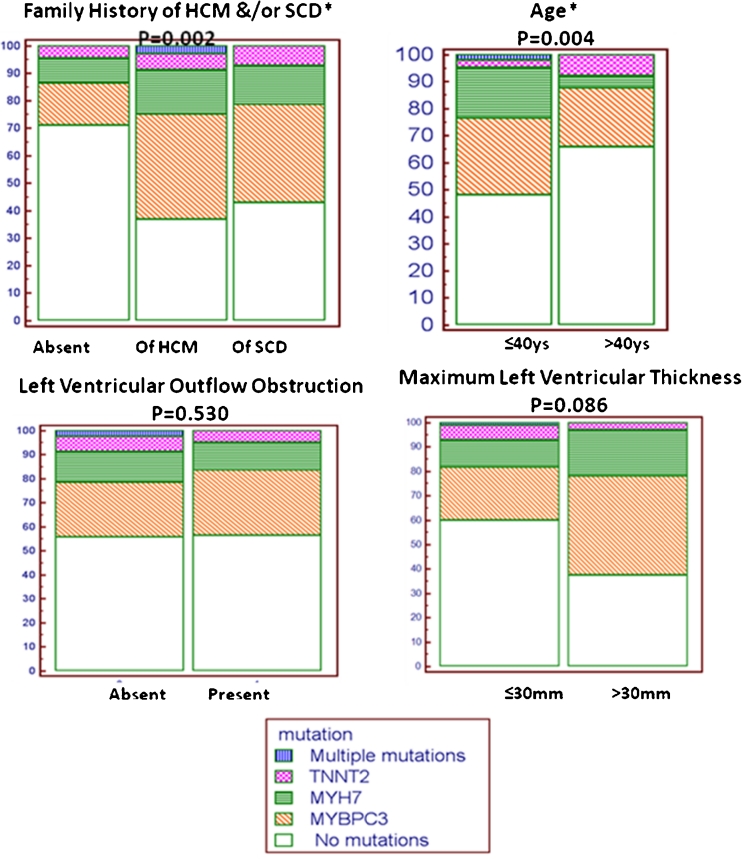
Univariate analysis of clinical parameters as predictors of positive sarcomeric genetic yield in Egyptian HCM patients using chi-square test (MedCalc-version 12.2.1) *Statistically significant association at *p* value <0.05

The clinical parameters comprised of presence of familial history of HCM and/or occurrence of sudden cardiac death (SCD) in the family, age of presentation at a cutoff ≤40 years, and presence/absence of LV outflow obstruction and maximum LV wall thickness being categorized into two groups (≤30 mm and >30 mm) were assessed in relevance to detection of positive mutation. It was observed that the positive genetic yield in any of the three sarcomeric genes was significantly associated with presence of family history of HCM and/or occurrence of SCD (*p* = 0.002) and with young age of presentation at ≤40 years (*p* =0.004) (Fig. 4). There was an observed trend of presence of mutation in association with increased LV maximum thickness (>30 mm), but this did not reach significance (*p* = 0.08). There was no statistically significant difference in the LV thickness among the genotype-positive cases of the three sarcomeric genes (using analysis of variance (ANOVA) test, *p* = 0.36, Table 6).

**Table 6:** Comparison of LV maximum thickness among the genotype positive cases of the three sarcomeric genes

	*MYBPC3*	*MYH7*	*TNNT2*	Double_hits
Mean (SD)	2.59 (0.61)	2.57 (0.70)	2.17 (0.42)	2.39 (0.88)
ANOVA	1.08			
*P* value	0.36			

There was also no statistically significant difference observed between the genopositive and genonegative patients with respect to initial clinical presentation with regards to severe degree of dyspnea (NYHA class III/IV), chest pain, and syncope (24 vs. 29 *p* = 0.39, 51 vs. 75 *p* = 0.95, and 19 vs. 22 *p* = 0.37, respectively). The overall prevalence of atrial fibrillation was not significantly different between both groups (*p* = 0.80). It was observed that prior cardiac arrests in this cohort (Table 1) occurred only in genopositive patients. As for incidence of other events as cardiac death and stroke, they were similar between the two groups. It is difficult at present to assess a correlation between adverse major cardiac events and genetic status due to the relatively short follow-up duration (median 38.8 months, range 30–49 months). Assessment of genotype–phenotype correlation is still under investigation.

## Discussion

This study represents the first report of comprehensive screening of *MYBPC3*, *MYH7*, and *TNNT2* in 192 Egyptian HCM patients, of whom 78 were familial (40.6 %). Initial screening of commonly involved sarcomeric genes is a justifiable first line approach of HCM genotype analysis [12, 14]. The frequency of positive sarcomeric genotyping detected among the Egyptian HCM index patients (40 %) is comparable to the frequency reported by Van Driest et al. in an earlier study [6], in which eight sarcomeric genes were analyzed in 389 HCM patients (38 % were genopositive). Also, recently, Curila et al. reported a similar frequency (40 %) analyzing four sarcomeric genes (*MYBPC3*, *MYH7*, *TNNT2*, and *TNNI3*) in 100 HCM patient [37]. However, the proportion of genetic yield varied among different population cohorts and ranged from >50 % in France and Italy [12, 14] to <30 % in Sweden [15]. This variation in frequency of positive genetic yield may probably relate to the proportion of familial cases included in the different cohorts. The positive sarcomeric genetic yield detected in the present cohort was significantly associated with presence of family history of HCM and/or occurrence of sudden cardiac death (*p* = 0.002) and with young age of presentation (*p* = 0.004).

*MYBPC3* was the most commonly involved sarcomeric gene (24 %) and was twice as common as *MYH7* (12 %), and *TNNT2* was the least commonly involved in the present cohort. *MYBPC3* and *MYH7* accounted for 87 % of all genotype-positive patients. A similar genotype pattern was also observed in several European HCM cohorts [12, 14, 25, 37].

HCM is considered a highly heterogeneous disease not just at the phenotype but also showing marked genetic heterogeneity. This is reflected as a high frequency of novel mutations reported in different population studies [6, 12, 14] limiting the possibility of screening for specific genes or particular mutations in research and clinical practice. However, different ethnic cohorts including the present study revealed repetitive particular genotype association that may relate to a founder effect [14, 38–40], highlighting the importance of genetic screening in different populations.

A high proportion of the mutations detected in the present cohort were novel (65 %) and assessment of their potential pathogenicity was undertaken based on the practice guidelines for interpretation of unclassified variants in clinical molecular genetics, and the overall conclusion was based on more than a single line of evidence [41]. Accordingly, novel variants in the present study were classified as: uncertain, likely pathogenic, and pathogenic. This molecular stratification of variants was always considered in genetic counseling. An example is of a family pedigree with a novel variant (*MYH7*: Asp309Asn) in index patient (PA8). This novel variant, according to in silico bioinformatic analysis, was predicted as probably damaging by PolyPhen-2 (score 0.963) and was considered as unlikely to be damaging by other in silico tools (Table 4). It has not been detected in 200 chromosomes of matched healthy controls and was tested for cosegregation within the family. The mutation was detected in two sibs who were phenonegative by standard echocardiographic screening. Thus, confirmation of pathogenicity is not possible at the current stage; however, follow-up of the phenonegative sibs is warranted since the mutation pathogenicity is uncertain and reduced penetrance is a possibility.

Single nucleotide substitution was the most commonly encountered mutation type (51/62, 82 %). These substitutions were analyzed in context of the reference GenBank sequence of the three sarcomeric genes to determine whether deamination of five methyl cytosine and transition to thymine represented a common cause of mutagenesis in sense or antisense DNA strands of sarcomeric genes in our HCM cohort [42–45]. It was found that C > T and G > A transitions constituted 78 % (21/27) of the substitutions occurring within the CpG dinucleotides in the present cohort (as shown in Table 5). This is in agreement with the frequency of C > T and G > A transition reported by Meures and Mealey [46] in substitution mutations found in HCM in the literature (Table 7).

**Table 7:** Frequency of different substitution modes within CpG in the present cohort in comparison to previously reported mutations in the 3 analyzed sarcomeric genes

Mode of substitution in CpG	Present study	Previously reported CpG substitutions^a^
G > A	15/27 = 55.6 %	113/251 = 45 %
C > T	6/27 = 22.2 %	56/251 = 22.3 %
C > A	3/27 = 11.1 %	13/251 = 5.2 %
C > G	0/27 = 0 %	18/251 = 7.2 %
G > C	2/27 = 7.4 %	30/251 = 12.0 %
G > T	1/27 = 3.7 %	21/251 = 8.4 %

^a^[46]

This observation probably explains the theory behind some mutation recurrence in several HCM cohorts of different ethnicity: such as the functionally characterized [47] Arg92codon mutation of TNNT2 reported in several studies [34, 35, 48], the E258K mutation of MYBPC3 reported with a founder effect among the Italian population [14], and the Ala797Thr reported with a possible founder effect in South Africa [49]. All such missense mutations involve CpG sites and are, therefore, likely to be hypermutable spots and hence, were also detected in other studies including the present. The hypothesis of considering codon Arg92 as a putative hot spot with high mutation rate rather than recurrence among different populations due to a common founder effect was earlier proposed by Forissier et al. [48]. It is worth mentioning that other reported founder mutation resulting from deletion or insertion such as the Dutch 2373insG (Q791fs) in MYBPC3 [50] was not detected in the current study. The high mutability at the CpG sites within the sarcomeric genes probably explains also the high frequency of novel mutations detected in different studies [12, 14, 25, 37] including that observed in the present study (65 %).

## Conclusion and Future Directions

This study is the first to provide genetic data of HCM among Egyptians. Positive genotype was detected in 40 % of our cohort. Expanding the genetic screening analysis through use of next-generation sequencing technology to enable simultaneous analysis of a broad panel of inherited cardiovascular disease-related genes is expected to increase the genetic-positive yield. Molecular characterization of HCM in different ethnic populations with different genetic backgrounds will aid in global comparative studies helping to understand the pathophysiology and heterogeneity of HCM. The true value of these studies lies in their translation to the clinical setting and their utilization towards improved HCM diagnosis, prognosis, and treatment [51, 52]. It is anticipated that the molecular screening of HCM patients and their family members is likely to improve the management of HCM in Egypt.

## Appendix 1

Variants detected in Egyptian controls in comparison with European Americans and African Americans from Exome Variant Server database

**Table:**

Gene	Ex./In.	Mutation name (c.DNA)	Amino acid change	MAF/minor allele count in EVS			dbSNP
				Egyptian	European American	African American	
*MYBPC3*	Ex.4	c.472G > A	V158M	T = 0.06/12	T = 0.0864/718	T = 0.0171/69	rs3729986
*MYBPC3*	In.5	c.506-12delC	NA	Del. = 0.23/46	–	–	rs11570050
*MYBPC3*	In.11	c.927-46G > C	NA	G = 0.005/1	G = 0.0001/1	G = 0.016/62	rs73451796
*MYBPC3*	In.13	c.1223 + 29G > A	NA	T = 0.01/2	T = 0.1174/958	T = 0.0366/ 141	rs11570078
*MYBPC3*	In.14	c.1227-19C > A	NA	T = 0.02/4	–	–	–
*MYBPC3*	In.14	c.1227-32T > A	NA	T = 0.005/1	–	–	–
*MYBPC3*	In.16	c.1458-26C > G	NA	C = 0.005/1	C = 0.0/0	C = 0.0112/47	rs11570081
*MYBPC3*	In.22	c.2149-5C > T	NA	A = 0.005/1	–	–	rs36211722
*MYBPC3*	In.22	2148 + 39T > C	NA	G = 0.005/1	–	–	
*MYBPC3*	In.23	c.2308 + 18C > T	NA	A = 0.015/3	–	–	–
*MYBPC3*	In.26	c.2737 + 12C > T	NA	A = 0.025/5	A = 0.0257/206	A = 0.0513/192	rs3729936
*MYH7*	In.11	c.999 + 44T > C	NA	G = 0.005/1	G = 0.0886/762	G = 0.4017/1770	rs3729810
*MYH7*	In.16	c.1888 + 23G > A	NA	T = 0.02/4	–	–	–
*MYH7*	In.20	c.2287-43C > T	NA	A = 0.005/1	A = 0.0001/1	A = 0.0/0	–
*MYH7*	In.23	c.2923-18G > A	NA	T = 0.02/4	T = 0.0009/8	T = 0.1512/666	rs7157087
*MYH7*	In.27	c.3337-3dup	NA	Dup. = 0.08/16	–	–	rs45504498
*MYH7*	In.28	c.3853 + 27T > A	NA	T = 0.01/2	T = 0.3628/3120	T = 0.6239/4406	rs2277475
*MYH7*	In.32	c.4520-3C > T	NA	A = 0.005/1	–	–	–
*MYH7*	Ex.32	c.4472C > G	S1491C	C = 0.005/1	C = 0.0115/99	C = 0.003/13	rs3729823
*TNNT2*	In.2	c.41 + 75C > G	NA	C = 0.005/1	C = 0.0/0	C = 0.0275/38	rs10920185
*TNNT2*	In.2	c.41 + 86C > A	NA	T = 0.005/1	–	–	–
*TNNT2*	In.2	c.42-20G > A	NA	T = 0.025/5	T = 0.0001/1	T = 0.035/154	rs45561443
*TNNT2*	In.3	c.53-7_11del	NA	Del. = 0.41/82	–	–	rs45533739
*TNNT2*	In.6	c.133 + 12G > A	NA	T = 0.025/5	T = 0.0003/3	T = 0.0502/221	rs45580032
*TNNT2*	EX.6	c.83C > T	A28V	A = 0.005/1	–	–	–
*TNNT2*	In.8	c.203 + 7G > A	NA	T = 0.005/1	–	–	–
*TNNT2*	In.8	c.203 + 67G > A	NA	T = 0.01/200	T = 0.9991/3179	T = 0.9234/1384	rs1573230
*TNNT2*	In.9	c.264 + 7G > A	NA	T = 0.005/1	T = 0.0003/3	T = 0.0311/137	rs45490292
*TNNT2*	Ex.15	c.740A > G	K247R	C = 0.03/6	–	–	–
*TNNT2*	In.15	c.781-61A > G	NA	C = 0.02/4	–	–	
*TNNT2*	In.15	c.781-33C > T	NA	A = 0.025/5	A = 0.102/877	A = 0.4639/2044	rs2275863
*TNNT2*	In.16	c.821 + 22C > T	NA	A = 0.005/1	A = 0.0001/1	A = 0.0402/177	rs45495692
*TNNT2*	In.16	c.821 + 43C > T	NA	A = 0.01/2	A = 0.0003/3	A = 0.032/141	rs45570832
*TNNT2*	In.16	c.821 + 46C > T	NA	A = 0.015/3	A = 0.0283/243	A = 0.0057/25	rs45576635

Alleles frequency of European and African Americans were obtained from the Exome Variant Server (EVS) [36]

*MYBPC3* NM_000256.3, *MYH7* NM_000257.2, *TNNT2* NM_001001430.1, *Ex* exon, *In* intron

## Abbreviations

BA: Bibliotheca Alexandrina
ECG: Electrocardiogram
EVS: Exome Variant Server
HCM: Hypertrophic cardiomyopathy
MYF: Magdi Yacoub Foundation
SCD: Sudden Cardiac Death
